# Thoracoscopic posterior tracheopexy during primary esophageal atresia repair ameliorate tracheomalacia in neonates: a single-center retrospective comparative cohort study

**DOI:** 10.1186/s12893-022-01738-1

**Published:** 2022-07-25

**Authors:** Akihiro Yasui, Akinari Hinoki, Hizuru Amano, Chiyoe Shirota, Takahisa Tainaka, Wataru Sumida, Kazuki Yokota, Satoshi Makita, Masamune Okamoto, Aitaro Takimoto, Yoichi Nakagawa, Hiroo Uchida

**Affiliations:** Department of Pediatric Surgery, Nagoya University Graduate School of Medicine, 65 Tsurumai-cho, Showa-ku, Nagoya, Aichi 466-8550 Japan

**Keywords:** Esophageal atresia, Tracheomalacia, Thoracoscopic posterior tracheopexy, Primary esophageal atresia repair

## Abstract

**Background:**

Esophageal atresia (EA) is often associated with tracheomalacia (TM). The severity of TM symptoms varies widely, with serious cases requiring prolonged respiratory support and surgical treatment. Although we performed thoracoscopic posterior tracheopexy (TPT) during primary EA repair to prevent or reduce the symptoms of TM, few studies have investigated the safety and effectiveness of TPT during primary EA repair. Therefore, this study aimed to evaluate the safety and efficacy of TPT in neonates.

**Methods:**

We retrospectively reviewed the records of all patients diagnosed with TM who underwent primary thoracoscopic EA repair between 2013 and 2020 at the Nagoya University Hospital. Patients were divided into two groups: TPT (TPT group) and without TPT (control group). TPT has been performed in all patients with EA complicated by TM since 2020. We compared patient backgrounds, surgical outcomes, postoperative complications, and treatment efficacy.

**Results:**

Of the 22 patients reviewed, eight were in the TPT group and 14 were in the control group. There were no statistically significant differences in the surgical outcomes between the groups (operation time: *p* = 0.31; blood loss: *p* = 0.83; time to extubation: *p* = 0.30; time to start enteral feeding: *p* = 0.19; time to start oral feeding: *p* = 0.43). Conversion to open thoracotomy was not performed in any case. The median operative time required for posterior tracheopexy was 10 (8–15) min. There were no statistically significant differences in postoperative complications between the groups (chylothorax: *p* = 0.36; leakage: *p* = 1.00; stricture: *p* = 0.53). The respiratory dependence rate 30 days postoperative (2 [25%] vs. 11 [79%], *p* = 0.03) and the ratio of the lateral and anterior–posterior diameter of the trachea (LAR) were significantly lower in the TPT group (1.83 [1.66–2.78] vs. 3.59 [1.80–7.70], *p* = 0.01).

**Conclusions:**

TPT during primary EA repair for treatment of TM significantly lowered respiratory dependence rate at 30 days postoperative without increasing the risk of postoperative complications. This study suggested that TPT could improve TM associated with EA.

## Background

Esophageal atresia (EA) is often associated with tracheomalacia (TM). It has been reported that 15–79% of patients after EA repair have symptomatic TM [1, 2]; therefore, early diagnosis and treatment of TM is important in this population. TM is defined as an excessive collapse of the airway during expiration, resulting in wheezing and a barking cough that may lead to fatal “dying spells” [3, 4]. In symptomatic cases, long-term respiratory support and conventional surgical treatments, such as tracheostomy and aortopexy, are required [4].

In the last decade, some studies have reported posterior tracheopexy as a surgical approach for the treatment of TM. This approach directly addresses posterior membranous intrusion, which is a major cause of airway collapse in many patients with TM. In a large case series (N = 98), Shieh et al. reported that children who underwent posterior tracheopexy showed improvement in clinical symptoms [5]. Recently, thoracoscopic posterior tracheopexy (TPT) during primary EA repair in neonates has been preliminarily reported [6]. TPT would be a less invasive and more meaningful therapeutic intervention if it could be performed at the same time as primary EA repair, while still being effective. However, the safety and effectiveness of TPT during primary EA repair have not been fully evaluated yet.

This study evaluated the safety and efficacy of this new approach for TM in patients who underwent TPT during primary EA repair.

## Methods

We conducted a single-center retrospective comparative cohort study at the Nagoya University Hospital. All patients diagnosed with TM who underwent primary thoracoscopic EA repair between August 2013 and December 2020 were eligible. Beginning in 2020, TPT was performed in all patients with EA complicated by TM. Patients with TM were divided into two groups: those who had undergone TPT (TPT group) and those who had not (control group). In our study we diagnosed TM if perioperative bronchoscopy identified “U” shaped cartilage indicating that the wider posterior membranes were more likely to intrude into the trachea lumen, and if collapse of more than half of the anteroposterior tracheal wall was observed during exhalation [4]. We conducted these tests in patients of both groups. We compared patient backgrounds, surgical outcomes, postoperative complications, and treatment efficacy between the two groups. The study was approved by the Ethics Committee of Nagoya University Hospital (Ref No. 2020-0589).

**Fig. 1:**
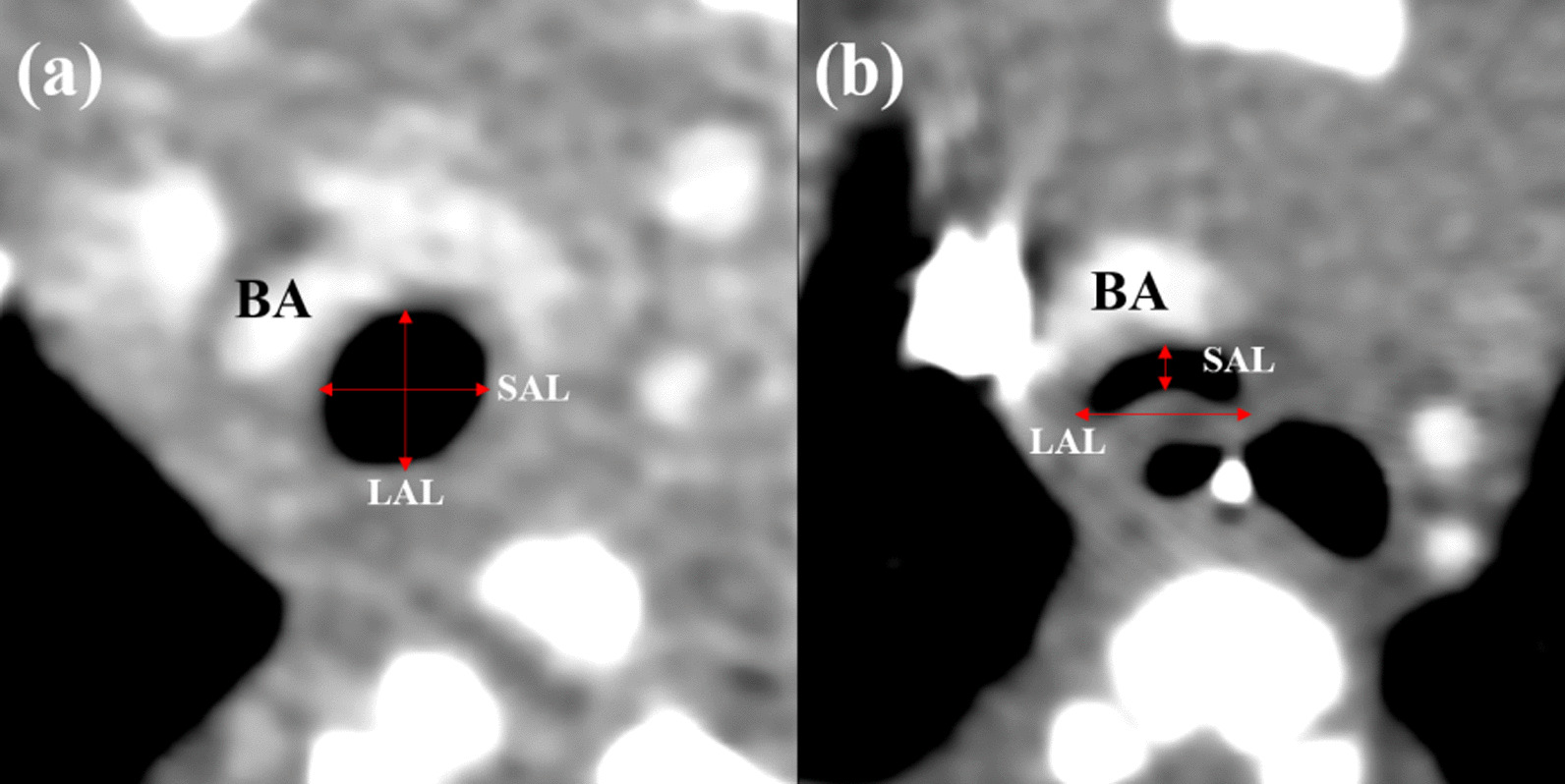
Computed tomography images indicating the calculation of LAR. At the level where the brachiocephalic artery crosses the trachea, LAR is calculated by dividing the long axis length of the trachea by the short axis length. **a** Neonate without tracheal collapse. **b** Neonate with tracheal collapse complicated by esophageal atresia. *BA* brachiocephalic artery, *LAL* long axis length of the trachea, *SAL* short axis length of the trachea

We used the ratio of the lateral and anterior–posterior diameter of the trachea (LAR) as an index of collapse of the posterior wall of the trachea [8]. Using contrast-enhanced computed tomography (CT) at the level where the brachiocephalic artery crosses the trachea, LAR was calculated by dividing the long axis length of the trachea by the short axis length (Fig. 1). We performed multiphasic scanning by multirow-detector helical CT, and thus we obtained multiple images in different phases. Therefore we could get the images that appeared closer at exhalation and inhalation. We measured LAR closer to the timing of exhalation. A larger LAR shows a more collapsed tracheal lumen, indicating a more vulnerable trachea. So, we used LAR as an indicator of TPT efficacy postoperatively and compared LAR values between the two groups. The median time to postoperative CT was postoperative day 26 (19–50; interquartile range). To evaluate the efficacy of TPT, we also examined the incidence of requiring respiratory support for the treatment of TM at 30 days postoperatively in the two groups, such as intubation or nasal continuous positive airway pressure therapy (CPAP). In addition, comparing LAR values between the groups with and without respiratory support at 30 days postoperatively, we evaluated the predictive performance of postoperative LAR values for long-term postoperative respiratory dependence by receiver operator characteristic curves (ROC) analysis.

### Operative technique

**Fig. 2:**
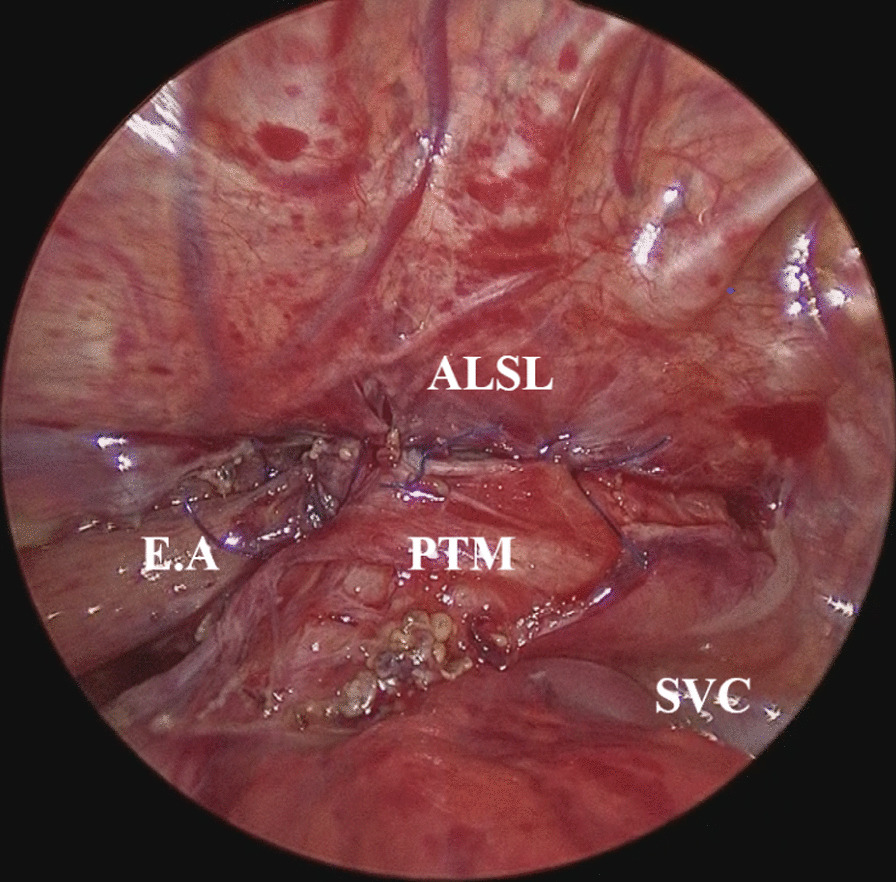
Surgical image of posterior tracheopexy. The posterior tracheal membrane was fixed to the anterior longitudinal spinal ligament by placing two sutures after esophageal anastomosis. *PTM* posterior tracheal membrane, *ALSL* anterior longitudinal spinal ligament, *EA* esophageal anastomosis, *SVC* superior vena cava

TPT during primary EA repair was performed with the patient placed in the three-quarter right prone position. The esophagus and trachea were dissected using 3-mm instruments. Three intercostal trocars, two 3-mm ports, and one 5-mm camera port were placed to access the right hemithorax. A CO_2_ pneumothorax was installed with a maximum pressure of 6 mmHg and a flow rate of 1 L/min. The tracheoesophageal fistula was closed using 4-0 absorbable sutures. The proximal pouch was then mobilized from the membranous posterior tracheal wall. The two ends of the esophagus were approximated, and end-to-end anastomosis using 5-0 absorbable sutures was performed. In one case of Gross type E EA, the tracheoesophageal fistula was dissected, and the trachea and esophagus were closed with interrupted sutures. The aimed height of the trachea for pexy was determined by intraoperative thoracoscopy or bronchoscopy, to identify the location where the posterior tracheal wall collapsed. Posterior tracheopexy was performed by placing one or two non-absorbable sutures (5-0 Prolene Polypropylene Suture, Ethicon Inc., Somerville, NJ, USA) that pulled the membranous posterior tracheal wall to the anterior longitudinal spinal ligament (Fig. 2). During this observation period, postoperative management such as antibiotic administration, timing of enteral nutrition, and chest drain removal, was standardized for all patients.

### Statistical analysis

Continuous variables are expressed as medians (interquartile range). The Mann–Whitney U test was used to compare continuous variables. Fisher’s exact probability test was used to analyze the differences between the discrete variables. Statistical significance was set at *p* < 0.05. All statistical analyses were performed using R software version 4.0.3 (R Foundation for Statistical Computing, Vienna, Austria). Receiver operator characteristic curves (ROC) analysis was used to evaluate the predictive performance of postoperative LAR values for long-term postoperative respiratory dependence.

## Results

### Patient characteristics

Between August 2013 and December 2020, 57 patients with EA underwent thoracoscopic repair. Twenty-two out of the 57 EA patients were suffering from TM: eight in the TPT group and 14 in the control group. There were two cases of TM that occurred after the age of 1 year, but these were excluded from this study. Sex, weight at surgery, and prematurity, did not differ significantly between the TPT and control groups (*p* = 0.66, *p* = 0.17 and *p* = 1.00, respectively; Table 1). There were no patients in both groups with ARDS, sepsis, or heart failure requiring inotropic drugs in relation to prolonged need for respiratory support. Median age at surgery was significantly different between the two groups (*p* < 0.01). However, this difference is because the TPT group included one patient with Gross type E EA who underwent surgery 20 days after birth.

**Table 1:** Comparison of patient characteristics variables, surgical outcomes, and postoperative complications between two groups

	TPT (n = 8)	Control (n = 14)	*p* value
Patient characteristics			
Male sex, n (%)	3 (38%)	8 (57%)	0.66
Age at surgery, (days), median (range)	2 (0–20)	1 (0–2)	< 0.01
Weight at surgery, (g), median (range)	2967 (1926–3337)	2446 (1678–3122)	0.17
Prematurity, n (%)	2 (25%)	4 (29%)	1.00
Surgical outcomes, median (range)			
Operation time, min	137 (119–180)	183 (73–280)	0.43
Blood loss, ml	1 (0–10)	1 (0–4)	0.83
Extubation, POD	1 (1–35)	2 (1–6)	0.30
Drain, POD	7 (4–8)	7 (5–12)	0.46
Enteral nutrition, POD	3 (1–7)	4 (3–7)	0.19
Oral feeding, POD	11 (7–40)	15 (6–45)	0.43
Postoperative complications, n (%)			
Leakage (%)	0 (0%)	1 (7%)	1.00
Chylothorax (%)	1 (13%)	0 (0%)	0.36
Anastomotic stricture (%)	1 (13%)	3 (21%)	0.53

*POD* postoperative day

### Surgical outcomes

Table 1 shows the surgical outcomes in both groups. There was no significant difference between the groups in terms of operation time (*p* = 0.31), blood loss (*p* = 0.83), time to extubation (*p* = 0.30), time to start enteral feeding (*p* = 0.19), and time to start oral feeding (*p* = 0.43). Conversion to open thoracotomy was not performed in any case. The median operative time required for posterior tracheopexy was 10 (8–15) min.

### Postoperative complications

There were no significant differences in postoperative complications between the groups (chylothorax: *p* = 0.36: leakage: *p* = 1.00; stricture: *p* = 0.53), as shown in Table 1. In the TPT group, one patient had both chylothorax and anastomotic strictures. The chylothorax was conservatively resolved, but the anastomotic stricture required balloon dilation.

### Efficacy of TPT

The respiratory dependence rate in the treatment for TM at 30 days postoperative was significantly lower in the TPT group than in the control group (*p* = 0.03; Table 2). In the control group, 10 patients required CPAP and one patient was intubated, while only 2 patients in TPT group required CPAP. In the control group, eight patients (57%) required additional surgical treatment, including tracheostomy in five patients (36%) and aortopexy in three patients (21%). In contrast, only one patient underwent tracheostomy in the TPT group. In all patients who underwent additional surgical procedures, these procedures were performed around 60 days after the initial surgery (details given in Table 3).

**Table 2:** The incidence of respiratory support at 30 days postoperatively

	TPT group (n = 8)	Control group (n = 14)	*p* value
Respiratory dependence rate, n (%)	2 (25)	11 (79)	0.03*
Intubation	0 (0)	1 (7)	1.00
CPAP	2 (25)	10 (72)	0.07
Surgical intervention, n (%)	1 (13)	8 (57)	0.07
Tracheostomy	1 (13)	5 (36)	0.35
Aortopexy	0 (0)	3 (21)	0.27

*Indicates statistical significance

**Table 3:** The postoperative time at which additional interventions were performed

	TPT group (n = 1)	Control group (n = 8)
Tracheostomy, (POD), median (range)	69	55 (31–60) (n = 3)
Aortopexy, (POD), median (range)	–	60 (44–81) (n = 5)

*POD* postoperative day

### Evaluation of the LAR

**Fig. 3:**
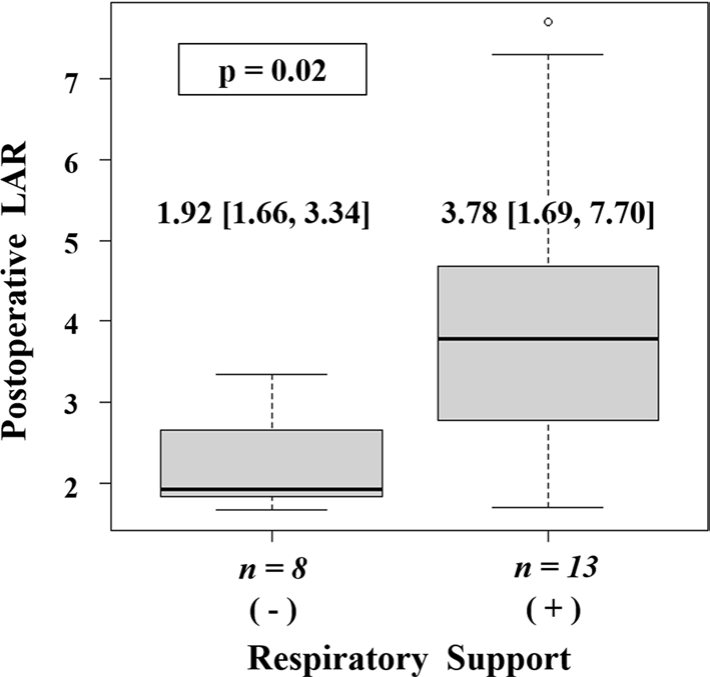
Comparison of postoperative LAR between two groups with or without respiratory support at 30 days

**Fig. 4:**
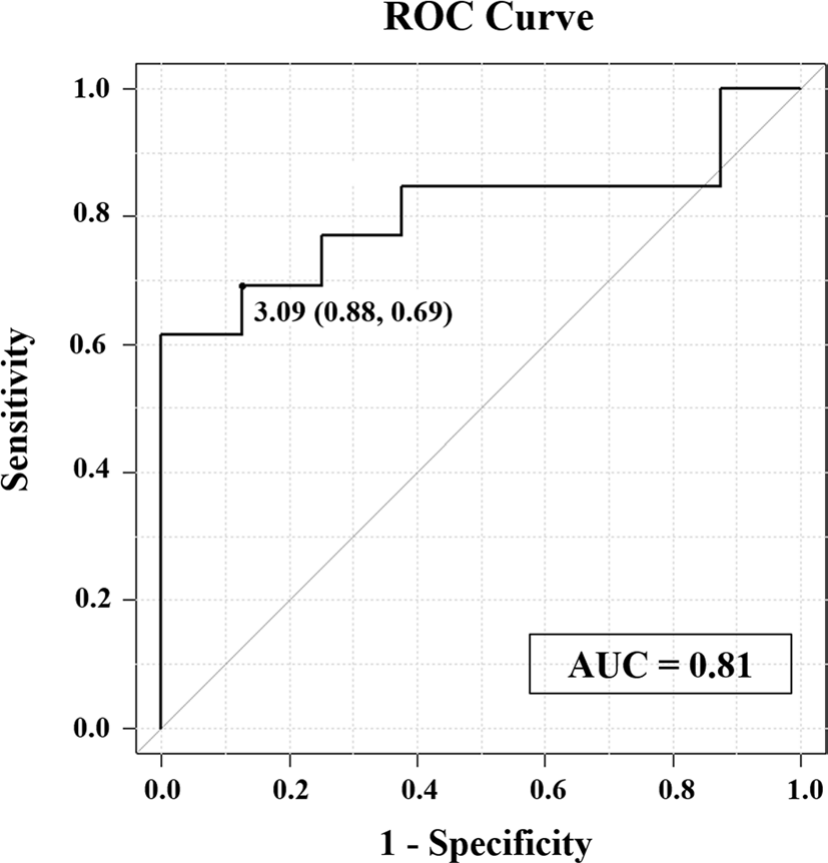
Evaluation of LAR as diagnostic indicator for TM requiring prolonged respiratory support after EA repair using ROC curve

Pre- and post-operative CT were done in 7 patients in the TPT group, and in 14 patients in control group. The value of postoperative LAR was significantly lower in the TPT group than in the control group (1.83 [1.66–2.78] vs 3.59 [1.80–7.70], *p* = 0.01, median [range]). Comparing the LAR between two groups with or without respiratory support at 30 days postoperatively (independent of the TPT procedure), LAR was higher in the former group (Fig. 3). At ROC analysis, the most accurate cut-off of the LAR to predict the need for long-term postoperative respiratory support was 3.09 (AUC 0.81; sensitivity 88%, specificity 69%, Fig. 4). One patient without respiratory support in the TPT group was excluded because his CT was not taken, and his LAR had not been measured.

## Discussion

Some studies have recently demonstrated the feasibility and efficacy of posterior tracheopexy as a treatment for TM [5, 9, 10]. However, all studies except one were conducted on patients aged one month or older. With the increasing demand for less invasive surgery and earlier treatment options, Tytgat et al. reported a case series detailing TPT during primary EA repair [6]. They also reported three cases of TPT after EA repair, indicating technical difficulty of mobilizing esophageal anastomosis and an apparent increase in postoperative complications.

To our knowledge, our study is the first to evaluate the safety and efficacy of TPT during primary EA repair in neonates. This study showed that additional TPT did not increase operation time, blood loss, or the risk of postoperative complications.

We observed a remarkably high rate of neonates needing respiratory support 1 month after EA repair without TPT. In our institution, CPAP is used relatively soon for patients with minor respiratory symptoms such as retractive breathing. In addition, we actively performed tracheostomy and aortopexy to prevent BRUE (brief resolved unexplained event). Therefore, the rate of need for respiratory support and additional surgical procedures appears to be high. Recently, several studies have reported that a significant number of TMs are associated with EA. One of them reported that out of 29 EA patients, 10 had mild/moderate TM and 13 had severe TM [2]. In addition, another study reported that 141 of 158 EA patients had TM [11]. However, none of these papers provided details on treatment such as respiratory support, tracheostomy, and aortopexy. Another article reported that 118 TM patients with EA underwent open tracheopexy over a 4-year study period [13]. This suggests that the number of TM patients requiring additional treatment may be larger than generally thought.

A previous report has recommended posterior trachea pexy prior to esophagus anastomosis to prevent compression of the left main bronchus by the esophagus [6]. At first, we performed the same procedure, but the sutured trachea interfered with the mobilization of the proximal pouch and the esophagus anastomosis. We felt TPT prior to esophageal anastomosis prevented the upper part of trachea from being subjected to tracheopexy. So, we decided to swap our surgical approach to performing TPT after anastomosis. In the latter, it is easier to observe the entire trachea while performing TPT. In addition, none of the patients in our study had clinical symptoms or CT findings indicating compression of the left main bronchus. However, it is necessary to accumulate and carefully monitor more cases, to further validate these observations.

One patient in the TPT group had both postoperative chylothorax and anastomotic stricture. Chylothorax after EA/TEF repair is rare and caused by injury to lymphatic vessels. However, adding posterior tracheopexy doesn’t require any extra dissection, so this procedure is unlikely to be a risk factor for postoperative chylothorax. There was no statistically significant difference in surgical outcomes and postoperative complications between the two groups; thus, our results indicate that TPT during primary EA repair is a safe procedure.

The respiratory dependence rate at 30 days postoperative was lower in the TPT group than in the control group. TM is caused by the collapse of the tracheal lumen during expiration; therefore, positive airway pressure treatment is important in mild or severe cases [7, 12]. Therefore, these results suggest that TPT can directly improve the collapse of the trachea. However, two patients in the TPT group required positive pressure ventilation 30 days postoperatively. One of the two patients required 5 cm H_2_O nasal continuous positive airway pressure at discharge, and the patient was completely weaned from respiratory support 9 months after surgery. The other patient with no chronic lung disease, who had long-segment tracheomalacia with bronchomalacia, did not show any improvement after TPT and required high-level positive end-expiratory pressure therapy; thus, tracheostomy and external fixation of tracheal airway stents were needed 13 months after initial surgery. This suggests that if the airway lesion is long, TPT alone is not sufficient and other surgical treatments should be considered. It is clear that TPT relieves symptoms caused by mild tracheomalacia that appear early in the postoperative period, and subsequently reduces respiratory support. On the other hand, our results did not indicate whether TPT is effective for the long-segment and long-term TM. To demonstrate this, long-term follow-up and accumulation of additional cases is needed.

As an evaluation of efficacy after tracheopexy, some studies have reported the usefulness of bronchoscopy to directly measure tracheal lumen patency [13, 14]. However, bronchoscopic evaluation is invasive and difficult to measure quantitatively. In our previous report, we had measured LAR in 51 normal patients who did not have tracheomalacia. This reported that the LAR is a good reflection of the degree of tracheal compression, and hence useful in judging the therapeutic effect of aortopexy [8]. Therefore, we assumed that the LAR value, which can be easily calculated from contrast-enhanced CT, would be a more quantitative index to evaluate tracheal lumen patency. According to our results, the postoperative LAR was significantly lower in the TPT group than in the control group, indicating an improvement in airway collapse. Moreover, the value of LAR was significantly higher in the group requiring prolonged respiratory support (Fig. 3). Our ROC analysis indicated that the postoperative LAR value showed moderate accuracy (AUC = 0.81). These results suggest that the LAR may be useful as an index of treatment efficacy and prognostic value for TM after EA repair.

We acknowledge that this study has several limitations, including the small number of patients and the retrospective design. Furthermore, the follow-up intervals were short, especially in the TPT group. Therefore, further research is needed to evaluate the efficacy and long-term prognosis of TPT.

## Conclusions

TPT during primary EA repair significantly lowered both the respiratory dependence rate in the treatment of TM at 30 days postoperative and the value of LAR, without increasing the risk of postoperative complications. This study suggests that TPT ameliorates TM associated with EA.

## Data Availability

The datasets generated and/or analyzed during the current study are available from the corresponding author on reasonable request.

## Abbreviations

EA: Esophageal atresia
TM: Tracheomalacia
TPT: Thoracoscopic posterior tracheopexy
LAR: The ratio of the lateral and anterior–posterior diameter of the trachea
CT: Computed tomography
CPAP: Continuous positive airway pressure therapy
ROC: Receiver operator characteristic curves
